# DPP8/9 inhibitors are universal activators of functional NLRP1 alleles

**DOI:** 10.1038/s41419-019-1817-5

**Published:** 2019-08-05

**Authors:** Kuo Gai, Marian C. Okondo, Sahana D. Rao, Ashley J. Chui, Daniel P. Ball, Darren C. Johnson, Daniel A. Bachovchin

**Affiliations:** 1 Chemical Biology Program, Memorial Sloan Kettering Cancer Center, New York, NY 10065 USA; 2 Tri-Institutional PhD Program in Chemical Biology, Memorial Sloan Kettering Cancer Center, New York, NY 10065 USA; 3 Pharmacology Program of the Weill Cornell Graduate School of Medical Sciences, Memorial Sloan Kettering Cancer Center, New York, NY 10065 USA

**Keywords:** Cell death, Cell death and immune response, Immune cell death, Monocytes and macrophages

## Abstract

Intracellular pathogenic structures or activities stimulate the formation of inflammasomes, which recruit and activate caspase-1 and trigger an inflammatory form of cell death called pyroptosis. The well-characterized mammalian inflammasome sensor proteins all detect one specific type of signal, for example double-stranded DNA or bacterial flagellin. Remarkably, NLRP1 was the first protein discovered to form an inflammasome, but the pathogenic signal that NLRP1 detects has not yet been identified. NLRP1 is highly polymorphic, even among inbred rodent strains, and it has been suggested that these diverse NLRP1 alleles may have evolved to detect entirely different stimuli. Intriguingly, inhibitors of the serine proteases DPP8 and DPP9 (DPP8/9) were recently shown to activate human NLRP1, its homolog CARD8, and several mouse NLRP1 alleles. Here, we show now that DPP8/9 inhibitors activate all functional rodent NLRP1 alleles, indicating that DPP8/9 inhibition induces a signal detected by all NLRP1 proteins. Moreover, we discovered that the NLRP1 allele sensitivities to DPP8/9 inhibitor-induced and *Toxoplasma gondii*-induced pyroptosis are strikingly similar, suggesting that DPP8/9 inhibition phenocopies a key activity of *T. gondii*. Overall, this work indicates that the highly polymorphic NLRP1 inflammasome indeed senses a specific signal like the other mammalian inflammasomes.

## Introduction

Inflammasomes are multi-protein complexes that form in response to intracellular pathogenic structures and activities^1,2^. For the formation of ‘canonical’ inflammasomes, a sensor protein detects its cognate stimulus, oligomerizes with the adapter protein ASC, and recruits and activates caspase-1. Caspase-1 then cleaves and activates the inflammatory cytokines IL-1β and IL-18 as well as gasdermin D (GSDMD)^3,4^. Once cleaved, the N-terminal fragment of GSDMD forms pores in the plasma membrane and induces an immunostimulatory type of cell death called pyroptosis^1,5–7^.

**Fig. 1:**
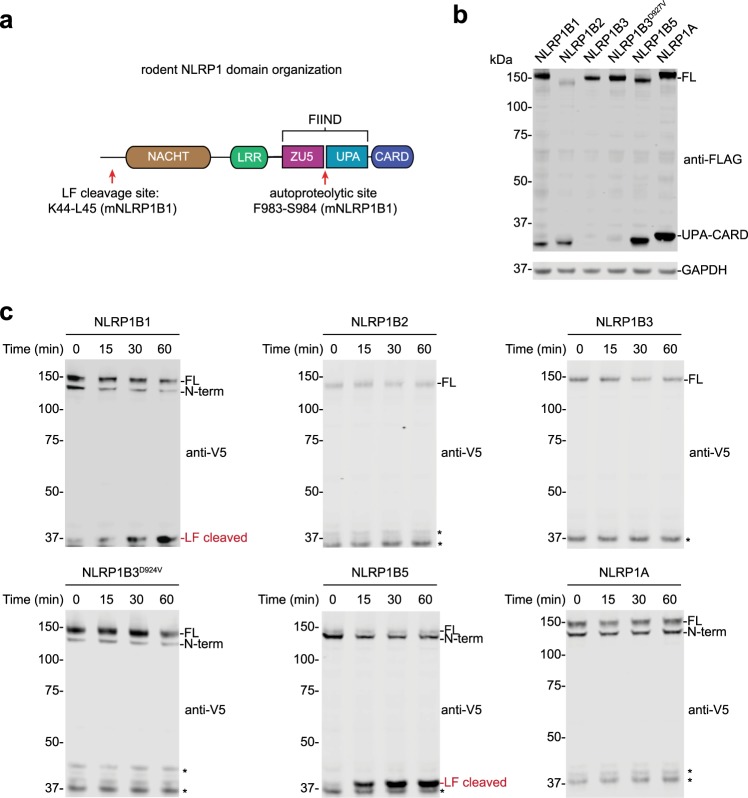
Mouse NLRP1 allele sensitivity to autoproteolysis and LT cleavage. **a** Domain organization of rodent NLRP1 proteins. The LF cleavage and FIIND autoproteolysis sites are indicated (residues correspond to the NLRP1B1 allele). The cartoon is not drawn to scale. **b** HEK 293T cells were transiently transfected with plasmids encoding the indicated C-terminally FLAG-tagged mouse NLRP1 alleles (2 µg, 48 h) before protein expression was evaluated by immunoblotting. FL full-length. **c** HEK 293T cells were transiently transfected with the indicated N-terminally V5-GFP-tagged mouse NLRP1 alleles (2 µg, 48 h). Cell lysates were then treated with LF (1 µg/mL) for the indicated times before reactions were quenched with 2× loading dye and LF cleavage evaluated by immunoblotting. Asterisks indicate background bands

NLRP1 is a nucleotide-binding domain and leucine-rich repeat-containing (NLR) sensor protein that forms a canonical inflammasome^1,2,8^. The human and rodent NLRP1 proteins both contain nucleotide-binding (NACHT), leucine-rich repeat (LRR), “function-to-find” (FIIND), and CARD domains (Fig. 1a), but the human protein also contains an N-terminal pyrin (PYD) domain. NLRP1 undergoes post-translational autoproteolysis between the “found in ZO-1 and UNC5” (ZU5) and “conserved in UNC5, PIDD, and Ankyrin” (UPA) sub-domains of the FIIND, creating two fragments that remain associated and auto-inhibited (Fig. 1a)^9–11^. Autoproteolysis is required for NLRP1 activation. Humans, but not rodents, also express CARD8, an NLRP1 homolog that has a similar ZU5-UPA-CARD structure that undergoes autoproteolysis but lacks the N-terminal domains^9^.

NLRP1 is highly variable among inbred rodent strains^8,12^. The mouse genome encodes three *NLRP1* paralogs, *Nlrp1a*, *Nlrp1b*, and *Nlrp1c*^8^, although *Nlrp1c* is predicted to be a pseudogene. *Nlrp1a* is relatively conserved^13^, but *Nlrp1b* is extremely polymorphic, with five alleles present in common inbred mouse strains (*Nlrp1b1*-*5*)^8^ (Table 1). Two of the NLRP1B proteins appear to be non-functional due to defective autoproteolysis (NLRP1B3) or truncation prior to the CARD (NLRP1B4)^8,11,14^. Mouse strains expressing NLRP1B1 do not express NLRP1A, but inbred strains with other NLRP1B alleles appear to express both paralogs^13^. Rats have one *Nlrp1* gene, and this gene is also polymorphic, with at least five different alleles present in common inbred rat strains^15^.

**Table 1:** Mouse NLRP1B allele sensitivity to anthrax lethal toxin and VbP

NLRP1B allele	Strains	Functional	Anthrax LT		VbP
			NLRP1 cleavage	Speck formation	Speck formation
1	129S1/SvimJ, BALB/cJ, C3H/HeJ, CBA/J, FVB/NJ, NON/LtJ, NZO/HILtJ, SWR/J	Yes	Yes	Yes	Yes
2	A/J, C57BL/6J, I/LnJ	Yes	No	No	Yes
3	AKR/J, NOD/LtJ, SJL/J	No (no autoproteolysis)	No	No	No
4	DBA/2J, P/J, SM/J	No (no CARD domain)	NT	NT	NT
5	CAST/EiJ	Yes	Yes	Yes	Yes

**Fig. 2:**
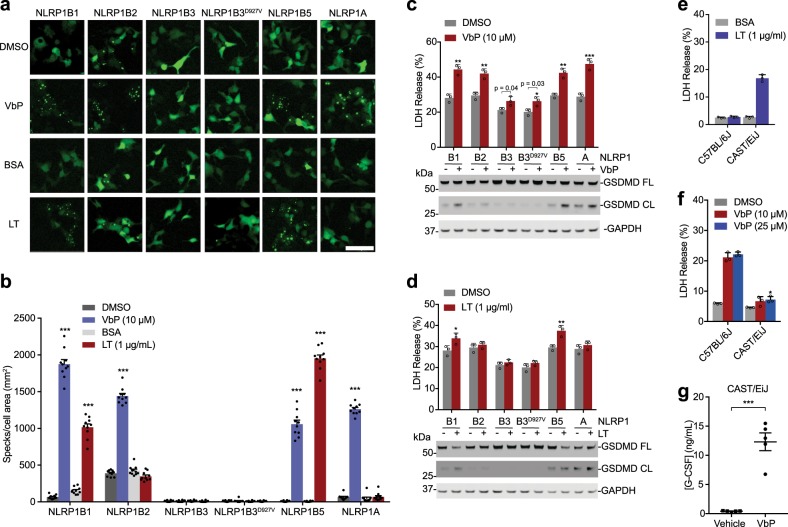
Mouse NLRP1 allele responsiveness to VbP and LT. **a**, **b** HEK 293T cells were transiently transfected with plasmids encoding the indicated FLAG-tagged mouse NLRP1 alleles and GFP-tagged ASC. After 24 h, cells were treated with BSA (1 µg/mL), DMSO, VbP (10 µM), or LT (1 µg/mL) for 6 h before ASC speck formation was assessed by fluorescence microscopy in 10 different regions. Representative images **a** and mean specks per cell area ± SEM **b** are shown. Scale bar is 100 µm. **c**, **d** HEK 293T cells stably expressing mouse CASP1 and mouse GSDMD were transiently transfected with plasmids encoding the indicated mouse NLRP1 alleles (0.01 µg). After 48 h, the cells were treated with VbP (10 µM, 24 h) **c** or LT (1 µg/mL, 6 h) **d** before cell death was evaluated by LDH release (top) and immunoblotting for GSDMD cleavage (bottom). Bar graphs are means ± SD of three biological replicates. **e**, **f** Primary BMDMs from C57BL/6J and CAST/EiJ mice were treated with LT (1 µg/mL, 6 h) **e** or the indicated concentration of VbP (48 h) **f** before LDH release was assessed. Data are means ± SD of triplicate wells and representative of at least three independent experiments. **g** Vehicle or VbP (20 µg/mouse) was administered intraperitoneally to CAST/EiJ mice, and serum G-CSF was assessed after 6 h by ELISA. Data are means ± SEM, *n* = 5 mice/group. *p < 0.05, **p < 0.01, ***p < 0.001 by two-sided Student’s *t*-test for vehicle vs. treated groups

The sensitivities of the five known NLRP1B alleles from inbred mouse strains to LT cleavage (Fig. 1c, Fig. S1), LT-induced speck formation (Fig. 2a, b), and VbP-induced speck formation are shown (Fig. 2a, b)

*NT* not tested

NLRP1 was the first protein discovered to form an inflammasome^16^, but a single cognate activation signal for all alleles, if one exists, has remained elusive. Anthrax lethal toxin (LT), the first discovered and best characterized NLRP1 trigger, activates only a subset of rodent NLRP1 alleles^8,15^. LT is a bipartite toxin comprised of lethal factor (LF), a zinc metalloprotease, and protective antigen (PA), a pore-forming protein that transports LF into the host cytosol. LF activates mNLRP1B alleles 1 and 5 (Table 1) and rNLRP1 alleles 1 and 2 (Table 2), but does not activate mNLRP1A, hNLRP1, or CARD8. LF directly cleaves each sensitive NLRP1 allele near its N-terminus (Fig. 1a)^17–19^, generating an unstable neo-N-terminus that is rapidly degraded by the “N-end rule” proteasome degradation pathway^20,21^. Because the C-terminus of NLRP1 is a separate polypeptide chain due to autoproteolytic cleavage, the CARD is not degraded by the proteasome, but is instead freed to form an inflammasome. *Shigella flexneri* IpaH7.8 ubiquitin ligase was recently shown to directly ubiquitinate the N-terminus of mNLRP1B1 (but not mNLRP1B2), resulting in its degradation and release of the C-terminal fragment^21^. In this way, NLRP1 alleles may potentially function as decoys for pathogen-encoded activities, with each allele perhaps tuned to sense different activities.

**Table 2:** Rat NLRP1 allele sensitivity to anthrax lethal toxin, *T. gondii*, and VbP

NLRP1 allele	Strains	Anthrax LT		*T. gondii*	VbP
		NLRP1 cleavage	MΦ pyroptosis	MΦ pyroptosis	MΦ pyroptosis (nM)
1	BN, WIS, SD, Dahl/SS	NT	Yes	Low^a^	257
2	CDF	Yes	Yes	Low^a^	88
3	ZUC	NT	No	NT	4
4	COP	NT	No	NT	3
5	LEW, WKY, SHR, SHR/Lj	No	No	High	3

**Fig. 3:**
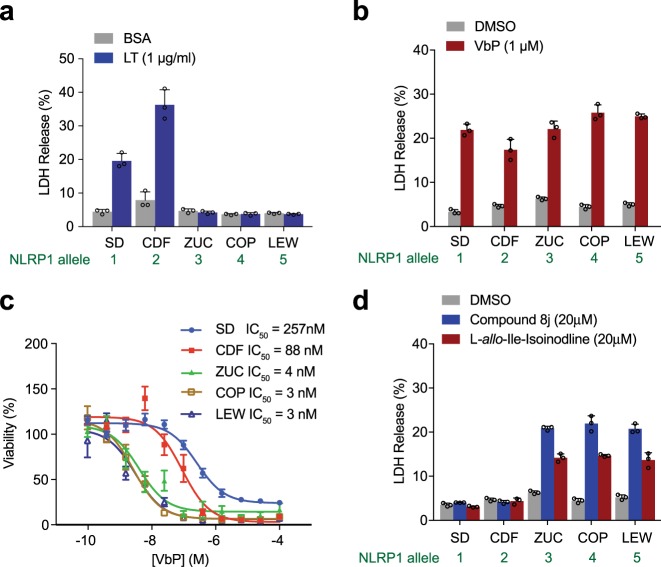
BMDMs from inbred rat strains are differentially sensitive to DPP8/9 inhibitors. **a** Rat BMDMs from the indicated rat strains were treated with LT for 8 h before cell death was evaluated by LDH release. The NLRP1 allele expressed by each macrophage is shown. SD Sprague Dawley, CDF Fischer, ZUC Zucker, COP Copenhagen, LEW Lewis. **b–d** Rat BMDMs were treated with the indicated concentration of VbP, compound 8j, or L-*allo*-Ile-isoindoline for 24 h before cell death was evaluated by LDH release **b**, **d** or CellTiter-Glo **c**. Data are means ± SD of triplicate wells and representative of at least three independent experiments

The sensitivities of the five known NLRP1 alleles from inbred rat strains to LT cleavage, LT-induced pyroptosis, *T. gondii*-induced pyroptosis, and VbP-induced pyroptosis. Data are from published reports^15,17,30,31^ and this study (IC_50_ values from Fig. 3b)

*NT* not tested

^a^*T. gondii* induces low levels of cell death and IL-1β release in these macrophages, but this response has not yet been definitely established as pyroptosis

We recently found that inhibitors of the host cell serine proteases DPP8 and DPP9 (DPP8/9), which cleave N-terminal dipeptides following proline from polypeptides^22–24^, also activate NLRP1B1 by inducing the proteasome-mediated degradation of the NLRP1B1 N-terminus^20,25^. The molecular details of this pathway remain unclear, but it does not involve the direct proteolysis of the N-terminal fragment like LT activation^26^. Intriguingly, preliminary data suggests that DPP8/9 inhibitors may be more universal NLRP1 activators than LT or IpaH7.8, as DPP8/9 inhibitors activate hNLRP1, hCARD8, and at least several mouse NLRP1 alleles^26–28^. However, it is not known if all NLRP1 alleles respond to DPP8/9 inhibition. In particular, the rat NLRP1 alleles have not yet been tested for DPP8/9 inhibitor responsiveness. Moreover, although prior studies have tested primary mouse macrophages for DPP8/9 inhibitor sensitivity^26,29^, the co-expression of mouse NLRP1A and NLRP1B prevented the unambiguous determination of which protein(s) was responsive.

Here, we show that DPP8/9 inhibitors are universal activators of all functional mouse NLRP1 alleles (i.e., those that have CARDs and undergo autoproteolysis). Notably, DPP8/9 inhibition activates the mouse NLRP1A protein, and is now the first known agent that activates the NLRP1A inflammasome. Similarly, we found that all rat NLRP1 alleles are sensitive to DPP8/9 inhibition, although the alleles differ profoundly in their relative sensitivities. On that note, *Toxoplasma gondii* was also recently shown to induce NLRP1-dependent pyroptosis in rat macrophages^30,31^. Although the mechanism of *T. gondii*-induced pyroptosis is unknown, the sensitivity of rat NLRP1 alleles to *T. gondii* matches their sensitivity to DPP8/9 inhibitors. Thus, it appears that DPP8/9 inhibition phenocopies some activity of this pathogen. More generally, these data suggest that all functional NLRP1 alleles, although quite distinct, do sense one universal stimulus: the cellular consequence of DPP8/9 inhibition.

## Materials and methods

### Cloning

cDNA encoding the mouse *Nlrp1b1* gene was cloned from RAW 264.7 macrophages. cDNA encoding the mouse *Nlrp1b3, Nlrp1b3-D927V*, and *Nlrp1b5* genes were obtained from R. Vance and J. Mogridge. cDNA encoding the full-length mouse *Nlrp1a* and *Nlrp1b2* were purchased from Genscript (OMu19634 and OMu00866D, respectively). As the *Nlrp1a* alleles in different inbred strains are highly similar between inbred mouse strains^13^, we only studied the C57BL/6J *Nlrp1a* sequence here. All *Nlrp1* cDNAs were subcloned into modified pLEX_307 vectors with N-terminal V5-GFP or C-terminal FLAG tags using Gateway technology (Thermo Fisher Scientific). cDNAs for mouse *Gsdmd*, mouse *Casp1*, and mouse *Pycard* were purchased from Origene. *Casp1* was subcloned into a modified pLEX_307 vector with a hygromycin resistance marker, *Gsdmd* was subcloned into a modified pLEX_307 vector with a C-terminal HA tag, and *Pycard* was subcloned into a modified pLEX_307 vector containing N-terminal V5-GFP and C-terminal FLAG tags using Gateway technology (Thermo Fisher Scientific).

### Reagents and antibodies

Val-boroPro^32^, compound 8j^33^, and L-a*llo*-Ile-isoindoline^34^ were synthesized according to previously published protocols. For cell culture experiments, Val-boroPro was resuspended in DMSO containing 0.1% TFA to prevent compound cyclization. Anthrax Lethal Factor (Catalog #172B) and PA (Catalog #171E) were purchased from List Labs, bortezomib from LC laboratories, and bestatin methyl ester from Sigma-Aldrich. Antibodies used include: anti-mouse caspase-1 (clone Casper-1, Adipogen), anti-GAPDH (clone 14C10, Cell Signaling Technology), anti-V5 (ab9116, Abcam), anti-GSDMD (ab209848, Abcam), and anti-FLAG (F1804, Sigma-Aldrich)

### Cell culture

HEK 293T and L Cells were purchased from ATCC and grown in Dulbecco’s modified Eagle’s medium (DMEM) with 10% fetal bovine serum (FBS) at 37 °C in a 5% CO_2_ incubator. Cell lines were tested for mycoplasma using the MycoAlert^TM^ Mycoplasma Detection Kit (Lonza).

### Cytokine stimulation in mice

The animal protocol was reviewed and approved by the Memorial Sloan Kettering Cancer Center Institutional Animal Care and Use Committee (IACUC). Sprague Dawley (SD:Stock #001), Fischer (CDFTM: Stock #002), Copenhagen (COP: Stock #286), Zucker (ZUC: Stock #186), and Lewis (LEW: Stock # 004) rats were purchased from Charles River laboratory. C57BL/6J (Stock #000664), CAST/EiJ (Stock #000928), NOD/ShiLtJ (Stock #001976), and AKR/J (Srock #000648) were purchased from Jackson laboratory.

For cytokine induction in C57BL/6J, CAST/EiJ, NOD/ShiLtJ, and AKR/J mice, 7–9-week-old female animals were administered 100 µL of vehicle (1 mM HCl in PBS, pH = 7.4) or Val-boroPro (20 µg/100 µL or 100 µg/100 µL) intraperitoneally. Val-boroPro was stored at 10× final concentration in 0.01 N HCl and diluted into PBS immediately before dosing. Serum was collected 6 h after dosing via retro-orbital collection and G-CSF levels were measured by Quantikine ELISA (R&D Systems). Sample size was based on the statistical analysis of previous experiments with vehicle-treated mice vs. Val-boroPro-treated mice^25,35^. No animals were excluded. The experiments were not randomized and the investigators were not blinded.

### LDH cytotoxicity assays

For LDH release experiments involving primary macrophages, mBMDM and rBMDM were isolated from the femurs and tibias of 7–9-week-old male and female mice and 7–12-week-old male rats. 1 × 10^7^ cells were plated on 10 cm Petri dishes. mBMDMs were differentiated in DMEM with 10% FBS and 20% L Cell medium for 6 days and rBMDMs in 30% L Cell medium for 7–9 days. Macrophages were scraped and reseeded at 0.25 × 10^6^ cells/well in 12-well plates in Opti-MEM and incubated overnight before treatment. Cells were then treated as indicated with compounds or LT. Supernatants were then harvested and analyzed for LDH activity using an LDH cytotoxicity assay kit (Pierce). For LDH release experiments in HEK 293T cells, we used cells stably expressing mCASP1 and mGSDMD described previously^20^. These cells were seeded at 0.5 × 10^6^ cells/well in six-well plates in standard growth medium, and transfected with 0.01 µg of the indicated *Nlrp1b* and 1.99 µg of a pLEX_307 plasmid containing *RFP* using the Fugene HD transfection reagent. After 48 h, cells were treated with Val-boroPro or LT as indicated, LDH release was assessed as described above. Lysates were normalized to 1 mg/mL using the DC Protein Assay kit (Bio-Rad), separated by SDS–PAGE, immunoblotted, and visualized using the Odyssey Imaging System (Li-Cor).

### ASC speck formation assay

HEK 293T cells were seeded into Lab-Tek II eight-well chambered coverglass plates at 2 × 10^4^ cells per chamber. After 48 h, the cells were transfected with 0.02 µg of plasmids encoding C-terminally FLAG-tagged mouse *Nlrp1* alleles, 0.01 µg of a plasmid encoding N-terminally V5-GFP-tagged mouse *Pycard*, and 0.37 µg of a plasmid encoding *RFP* using FuGene as the transfection agent, and given 24 h to express protein. Wells were then treated with the indicated agent for 6 h and imaged on a Zeiss Axio Observer.Z1 inverted widefield microscope using ×10/0.95NA air objective. For each chamber, 10 positions were imaged in the brightfield, RFP, and GFP channels. Data was analyzed using custom macro written in ImageJ/FIJI. Estimation of the total cell area within the field was obtained by measuring the standard area of fluorescence, counting the cellular area in the RFP channel filtered for intensity variance. The number of cells containing GFP-ASC specks was quantified by setting threshold values on the GFP channel, and performing the ‘Analyze Particles’ algorithm, size = 0–∞ and circularity = 0.50–1.00. The data was then exported to spreadsheet software, analyzed to compute the ratio of specks to the given cell area within the field, and visualized in GraphPad Prism 7 software.

### In vitro and in cell LF cleavage assays

HEK 293T cells were seeded at 0.5 × 10^6^ cells/well in six-well plates and transiently transfected with 2 µg of the indicated plasmids using FuGENE HD. For the in vitro assay, lysates were harvested after 48 h and normalized to 1 mg/mL using the DC Protein Assay kit (Bio-Rad). These lysates were then incubated with LF (1 µg/mL) for the indicated time periods at 25 °C quenched by boiling with 2 × SDS–PAGE sample buffer at the indicated times, separated by SDS–PAGE, immunoblotted, and visualized using the Odyssey Imaging System (Li-Cor). For the in cell assay, after 48 h after transfection cells were treated with LT (1 µg/mL, 6 h) before lysates were harvested for immunoblotting as described above.

### Cell Titer-Glo cell viability assay

Rat BMDMs (2000 cells/well) were plated in a white, 384-well clear bottom plates (Corning) in 25 μL final volume of media. Compounds were added using a pintool (a CyBio®-well Vario 96/250 384 channel simultaneous pipettor), and cells were incubated for 24 h at 37 °C. Assay plates were then removed from the incubator and allowed to equilibrate to room temperature on the bench top before addition of 10 µL of CellTiter-Glo reagent (Promega) according to the manufacturer’s instructions. Assay plates were shaken on an orbital shaker for 2 min and incubated at room temperature on the bench top for 10 min. Luminescence was then read using a Cytation 5 Cell Imaging Multi-Mode Reader (BioTek).

## Results

### Mouse NLRP1B3 is autoproteolysis-deficient and inefficiently cleaved by LT

At least one mouse NLRP1 protein, NLRP1B3, apparently does not undergo autoproteolysis^11^. We first wanted to verify this result, and confirm that the other mouse alleles do efficiently self-cleave. We therefore sub-cloned *Nlrp1a*, *Nlrp1b1*, *Nlrp1b2*, *Nlrp1b3*, and *Nlrp1b5* into an expression vector with a C-terminal FLAG tag. We did not study *Nlrp1b4* because it does not encode a CARD and therefore cannot form an inflammasome. We transiently transfected these vectors into HEK 293T cells and assayed for the generation of the FLAG-tagged UPA-CARD fragment by immunoblotting (Fig. 1b). As expected, all of the wild-type NLRP1 proteins, except NLRP1B3, underwent autoproteolysis. A D927V mutation in NLRP1B3 has been reported to restore at least some autoproteolysis, and although the NLRP1B3 D927V mutant protein did produce some detectable UPA-CARD fragment (Fig. 1b), the amount of this fragment was still considerably less than that produced by the other alleles.

LT induces pyroptosis only in mouse macrophages expressing NLRP1B1 or NLRP1B5^8^. LF has been shown to directly cleave NLRP1B1 and NLRP1B3, but not NLRP1B2, by immunoblotting^18,19,36^. It is widely assumed that LF cleaves NLRP1B5 based on macrophage sensitivity, but, to our knowledge, this has not been confirmed directly. We next wanted to verify the sensitivity of the mouse NLRP1 alleles to LF cleavage. We subcloned each allele into a mammalian expression vector with an N-terminal V5-GFP tag to enable visualization of the otherwise small LF-cleaved fragment by SDS–PAGE analysis. We then transiently transfected these vectors into HEK 293T cells, and treated lysates from these cells with the LF protease (Fig. 1c). As expected, LF-cleaved NLRP1B1 and NLRP1B5, but not NLRP1B2. Surprisingly, we did not see LF cleavage of NLRP1B3 or NLRP1B3 D927V (Fig. 1c), contrary to a previous report^18^. We observed similar results when we treated cells with LT directly (Fig. S1). We are currently unsure why LF cleaves NLRP1B3 less efficiently in our hands than in the previously reported study, but we speculate it may be due to differences in the activity of LF used or the exact NLRP1B3 constructs tested. Regardless, as NLRP1B3 is non-functional due to defective autoproteolysis, we did not consider its susceptibility to LF cleavage further.

### DPP8/9 inhibition activates all functional mouse NLRP1 alleles

We next wanted to determine which mouse alleles were activated by DPP8/9 inhibition. We transiently co-transfected vectors encoding each NLRP1 paralog together with a vector encoding GFP-tagged mouse ASC into HEK 293T cells. We then treated these cells with either the potent DPP8/9 inhibitor Val-boroPro (VbP) or LT, and assessed ASC speck formation by fluorescence microscopy (Fig. 2a, b). As expected, LT only induced speck formation in cells containing NLRP1B1 and NLRP1B5. VbP, in contrast, induced speck formation in cells containing NLRP1B1, NLRP1B2, NLRP1B5, and NLRP1A. We are unsure why NLRP1B2 spontaneously induced more ASC specks than the other alleles, but its lower level of expression (Fig. 1b) might indicate that it is less stable and thus more prone to uninduced proteasome-mediated activation. VbP did not activate the wild-type or mutant NLRP1B3 in this assay. To further verify these results, we transiently transfected the constructs encoding each NLRP1 allele into HEK 293T cells stably expressing CASP1 and GSDMD, and then treated these cells with VbP (Fig. 2c) or LT (Fig. 2d). As expected, LT only induced death in cells expressing NLRP1B1 and NLRP1B5. VbP, however, triggered death in cells expressing NLRP1B1, NLRP1B2, NLRP1B5, and NLRP1A. Interestingly, we also observed that VbP induced a small, but statistically significant amount of LDH release in cells expressing the NLRP1B3 proteins, suggesting that these proteins might not be entirely dead (Fig. 1b, c). However, no GSDMD cleavage was observed, consistent with their inactivity.

The above results establish VbP as the first known activator of the NLRP1A inflammasome in recombinant systems. As NOD/SHiLtJ and AKR/J mice express non-functional NLRP1B3 and functional NLRP1A^13^, any VbP-induced inflammasome activation in these mice can be largely ascribed to NLRP1A. Thus, to determine if VbP activates the NLRP1A inflammasome in vivo, we treated these mice strains with VbP. We found that VbP significantly increased the levels of serum G-CSF (Fig. S2), a previously used marker of VbP’s immunostimulatory activity^25,35^, indicating that VbP does indeed activate the NLRP1A inflammasome in vivo.

We previously reported that VbP (10 µM) did not induce significant cell death in bone-marrow-derived macrophage (BMDMs) from CAST/EiJ mice after 24 h. These BMDMs express NLRP1B5 and NLRP1A^13^, are susceptible to LT-induced death^8^, and, based on our data above, should be sensitive to VbP. We hypothesized that these CAST/EiJ BMDMs were less sensitive to VbP than C57BL/6J BMDMs, but were still likely responsive. We confirmed that CAST/EiJ, but not C57BL/6J, BMDMs are sensitive to LT (Fig. 2e), and then treated these BMDMs with high doses (25 µM) of VbP for 48 h. We found that VbP did induce slight cell death in CAST/EiJ BMDMs (Fig. 2f). We predicted that VbP-induced immune activation in mice might be easier to observe than macrophage pyroptosis, and indeed found that VbP induced high levels of serum G-CSF in CAST/EiJ mice (Fig. 2g). It is unclear why the CAST/EiJ BMDMs are considerably less sensitive to VbP than C57BL/6J BMDMs, but these mice differ in many complex phenotypes compared with other inbred strains^37^. Regardless, these data together unambiguously demonstrate that VbP activates mouse NLRP1B1, NLRP1B2, NLRP1B5, and NLRP1A.

### DPP8/9 inhibition activates all rat NLRP1 alleles

We next wanted to evaluate the sensitivity of inbred rat BMDMs, and thus their corresponding *Nlrp1* allele, to DPP8/9 inhibition. We isolated BMDMs from five inbred rat strains, one with each of the five known rat *Nlrp1* alleles^15^. As expected, LT induced cell death only in Sprague Dawley (SD, allele 1) and Fischer (CDF, allele 2) BMDMs (Fig. 3a). In contrast to LT, VbP induced cell death in BMDMs isolated from all five of the strains (Fig. 3b, c). Macrophages with alleles 3, 4, and 5 were extraordinarily sensitive to VbP, all with IC_50_ values <10 nM; macrophages with alleles 1 and 2 were much less sensitive, with IC_50_ values of 257 and 88 nM, respectively. To confirm that the toxicity observed in rat macrophages was due to DPP8/9 inhibition, we next treated these BMDMs with the more selective, albeit less potent DPP8/9 inhibitors compound 8j^33^ and L-a*llo*-Ile-isoindoline^34^ (Fig. 3d). As expected, we found that these inhibitors killed rat BMDMs with alleles 3, 4, or 5. Neither compound killed BMDMs with alleles 1 or 2 at the concentrations tested, consistent with their weaker potencies relative to VbP. Intriguingly, although sensitivities of rat BMDMs to VbP and LT are very different^15,17^, their sensitivities to VbP and *T. gondii* are strikingly similar, as discussed in detail below (Table 2)^30,31^.

### Proteasome inhibition blocks VbP-induced pyroptosis in rat BMDMs

**Fig. 4:**
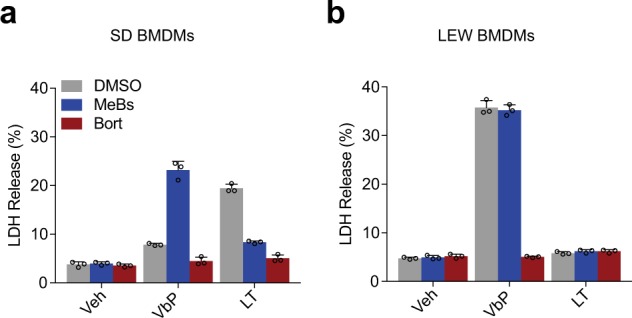
Proteasome inhibition blocks rat NLRP1 activation. **a**, **b** Primary BMDMs from SD **a** or LEW **b** rats were pretreated with bortezomib (bort, 1 µM) or bestatin methyl ester (MeBs, 2 µM) for 30 min prior to the addition of vehicle, VbP (2 µM), or LT (1 µg/mL) for an additional 8 h. Cytotoxicity was assessed by LDH release. Data are means ± s.d. of triplicate wells and representative of at least three independent experiments

We next asked if VbP-induced cell death in rat BMDMs requires proteasome activity. As in mice and humans, we found that bortezomib indeed blocked VbP-induced pyroptosis in SD (Fig. 4a, allele 1) and Lewis rat BMDMs (Fig. 4b, LEW, allele 5). It should be noted that these macrophages were only treated for 8 h (rather than 24 h, as in Fig. 3) because bortezomib is toxic over longer incubation times, and thus the overall magnitude of VbP-induced cell death in the SD macrophages was considerably lower in this experiment. We previously found that bestatin methyl ester (MeBs), a non-selective inhibitor of aminopeptidases, synergizes with VbP to induce more pyroptosis in both humans and mice^20^. We found that MeBs similarly synergized with VbP to induce profoundly more cell death in these SD macrophages (Fig. 4a, b). MeBs did not synergize with VbP in LEW macrophages, likely because these highly sensitive cells were already undergoing significant amounts of cell death. As expected, bortezomib and MeBs, which also inhibits the N-end rule pathway, blocked LT-induced pyroptosis in SD macrophages^20,38^ (Fig. 4a). Overall, these inhibitor sensitivity data indicate that VbP induces cell death via the same mechanism in rats, mice, and humans^20^.

## Discussion

**Fig. 5:**
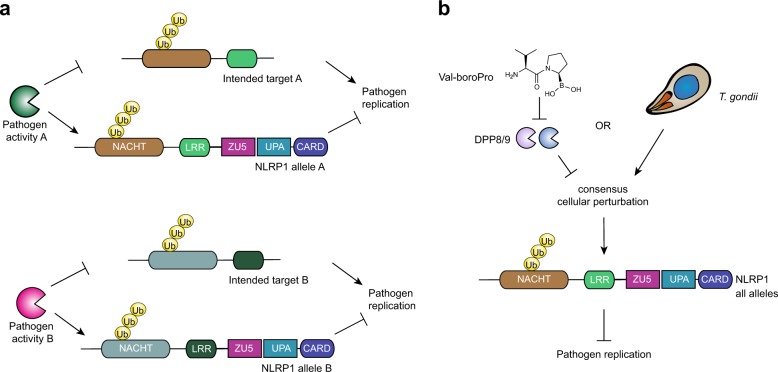
Potential models of NLRP1’s function. **a** The decoy hypothesis: pathogenic activities, like lethal factor, destroy host factors to aid pathogen replication. NLRP1 is a decoy for these host proteins, and its destruction results in an immune response and inhibition of pathogen replication. Different NLRP1 alleles might sense different pathogenic activities. **b** The consensus signal hypothesis: all NLRP1 alleles sense a specific cellular perturbation induced by DPP8/9 inhibitors, *T. gondii*, and likely other stimuli. The different NLRP1 alleles may have different tolerances for sensing this perturbation

The biological purpose of the NLRP1 inflammasome has not yet been fully established. Until recently, LT was the only known activator of NLRP1^8^. LT directly cleaves some rodent NLRP1 alleles (Fig. 1, Fig. S1)^17–19,36^, inducing the N-end rule-mediated degradation of their N-termini and releasing their C-termini to activate caspase-1^20,21^. This activation mechanism suggested one possibility for NLRP1’s function—that NLRP1 may act as “decoy target”, and not the intended target, of LF and possibly other pathogenic activities, and it uses its own stability to detect and mount immune responses to these activities (Fig. 5a). Consistent with this “decoy hypothesis”, the Vance laboratory showed that *S. flexneri* IpaH7.8 ubiquitin ligase directly ubiquitinates and activates mNLRP1B1^21^. An attractive feature of this model is that it is consistent with the extremely polymorphic nature of NLRP1, as, if correct, NLRP1 would be under intense and varied selection pressure applied by diverse pathogenic activities. Future studies are needed to determine if NLRP1 senses additional pathogen activities to further support this hypothesis.

An alternative (or perhaps an additional) possibility is that all NLRP1 alleles actually do detect one consensus activation signal, but that this activation signal has not yet been identified (Fig. 5b). Intriguingly, this study, together with previous reports^26–28^, have now demonstrated that DPP8/9 inhibition activates all of the known functional rodent and human NLRP1 proteins. However, the relative responsiveness of each allele to DPP8/9 inhibition does vary dramatically, especially in rats (Fig. 3). We speculate that DPP8/9 inhibition phenocopies some important pathogen activity, and perhaps the different alleles evolved to tolerate different levels or types of that particular activity. To our knowledge, no naturally occurring DPP8/9 inhibitors exist, but in theory this pathogen activity could direct DPP8/9 inhibition or somehow mimic a consequence of DPP8/9 inhibition.

On that note, inbred rat strains vary widely in their susceptibility to infection by *T. gondii*, and these differences are controlled by *Nlrp1* (Table 2)^30,31,39,40^. For example, LEW and SHR rats, which both contain *Nlrp1* allele 5, are highly resistant to *T. gondii* infection. In contrast, SD and Brown Norway (BN) rats, which contain allele 1, and CDF rats, which contain allele 2, are much more susceptible to *T. gondii* infection. *T. gondii* induces rapid pyroptosis in Lewis and SHR, but not in SD, BN, and CDF, rat macrophages^30,31^. These sensitivity data strongly suggest that rapid macrophage pyroptosis serves as a protective response to the parasite. It should be noted that *T. gondii* does induce some cell death and IL-1β release in the “pyroptosis-resistant” macrophages^30,31^, just much less than in the “pyroptosis-sensitive” macrophages. Interestingly, our study revealed that rodent macrophages are markedly similar in their relative sensitivities to DPP8/9 inhibitor and to *T. gondii*-induced pyroptosis (Table 2). We hypothesize that DPP8/9 inhibition phenocopies a key activity of *T. gondii*, and correspondingly that *T. gondii* is likely also a universal NLRP1 activator (Fig. 5b). Consistent with this premise, *T. gondii* induces also pyroptosis in C57BL/6J BMDMs (which express NLRP1B2 and NLRP1A)^30^, ectopic expression of mouse NLRP1B1 enhances *T. gondii*-induced pyroptosis in mouse BMDMs^30^, and *Nlrp1*-deficient mice have increased parasite loads and acute mortality rates after *T. gondii* challenge^41^. In humans, NLRP1 potentially plays a role in controlling *T. gondii* infection as well, as *NLRP1* polymorphisms are linked to human congenital toxoplasmosis^42^ and *T. gondii* induces inflammasome activation in human THP-1 monocytes^43^, but the exact roles of NLRP1, and potentially CARD8, remain to be established.

In summary, the biological function of NLRP1 has remained mysterious in large part due to a lack of known activators of the highly variable NLRP1 alleles. With this study, we have now established that DPP8/9 inhibition activates all functional NLRP1 alleles in rodents in addition to both NLRP1 and CARD8 in humans. These data suggest a key function of NLRP1 is likely to sense a specific pathogen-related signal, much like the other mammalian inflammasomes. We anticipate that future studies will build on these results to identify this key pathogenic signal and clarify the biological purpose of this enigmatic inflammasome.

## Supplementary information

Supplementary Information
