# Supplementary material for: DPP8/9 inhibitors are universal activators of functional NLRP1 alleles
Source: Cell Death Dis. 2019 Aug 5;10(8):587. doi: 10.1038/s41419-019-1817-5 (PMC6683174; doi:10.1038/s41419-019-1817-5)
Supplement: Supplementary file 1 — Supplementary Information [file 41419_2019_1817_MOESM1_ESM.pdf]

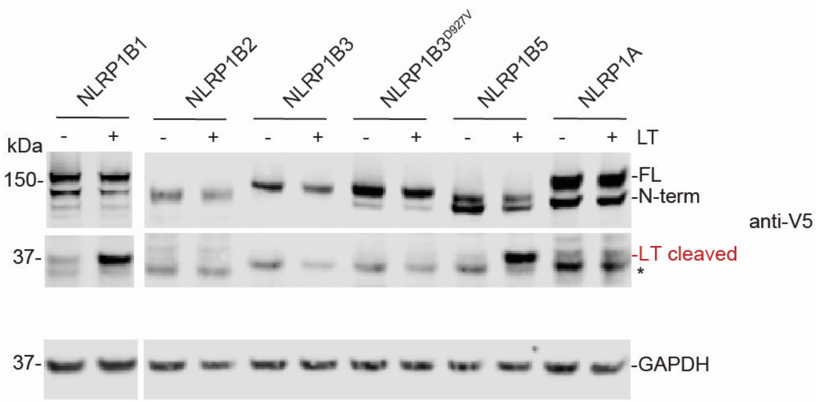

Fig S1. Mouse NLRP1 allele sensitivity to LT cleavage in cells. HEK 293T cells were transiently transfected with plasmids encoding the indicated N-terminally V5-GFP-tagged mouse NLPR1 alleles (2 µg, 48 h). Cells were then treated with LT (1 µg/mL, 6 h) before lysates were harvested and evaluated by immunoblotting. The asterisk indicates a background band. N-term, N-terminus.

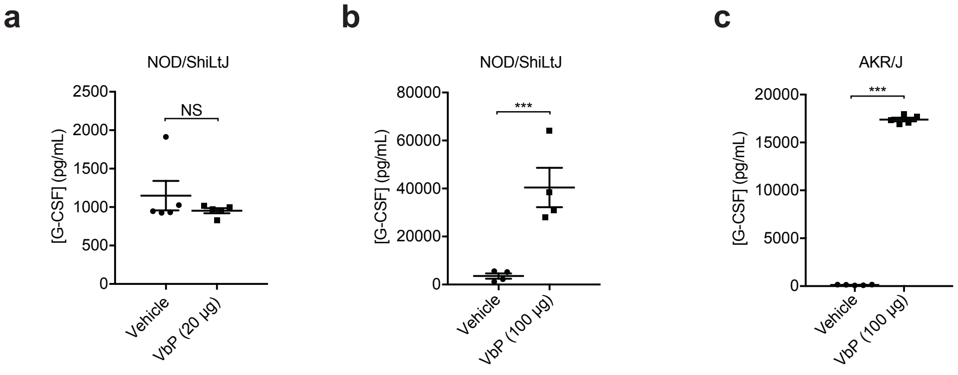

Fig S2. NOD/ShiLtJ and AKR/J are sensitive to VbP. (**a**-**c**) Vehicle or the indicated concentration of VbP was administered intraperitoneally to NOD/ShiLtJ (**a**,**b**) or AKR/J (**c**) mice. Serum G-CSF was assessed after 6 h by ELISA. Data are means ± SEM, n = 4-5 mice/group. ****p< 0.001 by two-sided Student's *t*-test for vehicle versus treated groups. NS, not significant. As NLRP1B3 is an inactive allele, the cytokine induction in these mice is likely due to activation of NLRP1A. NOD/SHiLtJ mice are less sensitive to VbP than C57BL/6J mice, as a lower dose (20 µg/mouse, i.p.) elevates G-CSF levels in C57BL/6J mice [22] but not in NOD/ShiLtJ mice (**a**).
